# The Influence of Smoking on Respiratory Function in Medical Students at the University of Medicine, Pharmacy, Science and Technology of Târgu-Mureș

**DOI:** 10.3390/biomedicines14010164

**Published:** 2026-01-13

**Authors:** Edith-Simona Ianosi, Renata-Ingrid Ianosi, Hajnal Finta, Raul-Alexandru Lefter, Anca Meda Văsieșiu, Dragoș Huțanu, Maria-Beatrice Ianosi

**Affiliations:** 1 Department of Pulmonology, George Emil Palade University of Medicine, Pharmacy, Science and Technology of Târgu-Mureș, 540139 Târgu Mureș, Romania; edith.ianosi@umfst.ro (E.-S.I.); dragos.hutanu@umfst.ro (D.H.); 2 George Emil Palade University of Medicine, Pharmacy, Science and Technology of Târgu-Mureș, 540139 Târgu Mureș, Romania; 3 Department of Public Health and Health Management, George Emil Palade University of Medicine, Pharmacy, Science and Technology of Târgu-Mureș, 540139 Târgu Mureș, Romania; hajnal.finta@umfst.ro; 4 Clinic of Pulmonology, County Hospital Mures, 540011 Târgu Mures, Romania; raul-alexandru-lefter@students.umfiasi.ro; 5 Department of Infectious Disease, George Emil Palade University of Medicine, Pharmacy, Science and Technology of Târgu-Mureș, 540139 Târgu Mureș, Romania; anca.vasiesiu@umfst.ro

**Keywords:** smoking, e-cigarettes, heated tobacco products, spirometry test, respiratory function parameters, small airways, lung function, medical students, sedentary lifestyle, physical activity

## Abstract

**Background:** Cigarette smoking remains one of the most important preventable causes of respiratory morbidity, exerting detrimental effects even in young adults. Medical students represent a particularly relevant population, as the lifestyle habits they adopt during their training years may influence both their personal health and professional credibility. **Methods:** We conducted a cross-sectional analysis of 264 medical students from the University of Medicine, Pharmacology, Science and Technology of Târgu-Mures, aged 18–30 years, stratified according to smoking status, type of tobacco product used, and lifestyle characteristics (athletic vs. sedentary). Standardized spirometry was performed to assess FVC, FEV_1_, FEV_1_/FVC ratio, PEF, and small airway flow parameters (MEF_25_, MEF_50_, MEF_75_). Statistical comparisons between groups were performed using *t*-tests, Mann–Whitney U tests, chi-square tests, and correlation analyses, with *p* < 0.05 considered statistically significant. **Results:** Smokers demonstrated significantly lower values for FEV_1_, PEF, and MEF parameters compared with non-smokers, confirming early functional impairment of both large and small airways. Within the smoking group, users of e-cigarettes or heated tobacco products exhibited more favorable FEV_1_ and small airway flow values than conventional cigarette smokers. However, differences in FVC were less pronounced. Significantly, athletes consistently outperformed their sedentary peers across all respiratory parameters, regardless of smoking status, with markedly higher FEV_1_, FVC, and MEF values and a lower prevalence of obstructive patterns. Cumulative smoking exposure (pack-years) was inversely associated with small airway function, whereas higher levels of physical activity were independently linked to a pronounced protective effect. **Conclusions:** Even in early adulthood, smoking is related to measurable declines in lung function, particularly affecting small airway dynamics. Although alternative products may appear less harmful than conventional cigarettes, they cannot be considered risk-free. Conversely, regular physical activity demonstrated a protective association in the case–control analysis, attenuating functional decline and supporting the preservation of long-term respiratory health. These findings underscore the importance of integrated prevention strategies in medical universities, combining smoking cessation initiatives with the systematic promotion of physical activity to safeguard the health of future physicians and reinforce their role as credible health advocates.

## 1. Introduction

Tobacco consumption remains one of the leading preventable contributors to global morbidity and premature mortality [1]. Emerging evidence indicates that even among university populations—particularly medical students who possess substantial theoretical awareness of tobacco-associated risks—smoking behavior continues to exert measurable adverse effects on respiratory function. Cross-sectional studies conducted in diverse geographical settings, including South Asian medical schools, have documented consistent impairments in peak expiratory flow and other spirometric indices among student smokers, demonstrating that functional deterioration is detectable even at relatively modest cumulative exposure levels [2,3,4,5,6]. These findings are particularly concerning, given that early adulthood is a formative period when lung function reaches its peak while long-term health behaviors are established. Consequently, even intermittent or low-intensity exposure to cigarette smoke during this developmental window may precipitate clinically relevant physiological alterations observable through standardized pulmonary-function testing [5,6,7].

Despite comprehensive curricular content on the harmful effects of tobacco, medical students remain susceptible to smoking initiation and continuation, a pattern shaped by the interplay of psychosocial stressors, peer influence, and an often-erroneous perception of invulnerability. Many students assume that brief experimentation or occasional use carries negligible risk and can be easily discontinued; however, measurable decrements in ventilatory performance among individuals with short smoking histories challenge the notion of a harmless “trial period,” indicating instead that functional impairment begins earlier than commonly perceived [5,6,7]. This misplaced belief in physiological resilience underscores the need for prevention strategies explicitly tailored to medical-student populations, whose future roles as health professionals depend heavily on both personal behavior and credibility in counseling patients.

Concurrently, evidence from the scientific literature increasingly underscores physical activity as a protective factor that may modulate the respiratory consequences associated with smoking. Regular engagement in exercise has been associated with enhanced ventilatory efficiency, reductions in systemic and airway inflammatory responses, and partial preservation of spirometric parameters in active smokers [1,8,9,10,11,12]. For medical students, structured physical activity may also offer benefits beyond physiological protection, including improved stress management, emotional regulation, and reduced smoking cravings, thereby supporting efforts toward cessation or reduction [10]. These multidimensional effects position physical activity as a potentially valuable adjunct in comprehensive university-based tobacco-control strategies.

At the physiological level, the detrimental impact of smoking is mediated through a complex mixture of toxic constituents present in cigarette smoke. Nicotine promotes addiction through neurobehavioral reinforcement pathways, carbon monoxide diminishes oxygen-transport capacity by forming carboxyhemoglobin, and reactive oxygen species contribute to oxidative stress and tissue injury. Furthermore, the deposition of tar particulates within the airway epithelium impairs mucociliary clearance and perpetuates chronic inflammation. These interacting mechanisms accelerate airway remodeling and functional decline, leading to reductions in FEV_1_, FVC, and expiratory flow rates—changes that are routinely identified even among young adults with relatively limited smoking exposure [5,6,7].

Spirometry remains the reference standard for detecting early alterations in pulmonary function, and studies among medical students consistently demonstrate reductions in FEV_1_, FVC, and various flow parameters in association with both active smoking and cumulative exposure [2,3,4,5,6,7]. Longitudinal data further suggest that regular physical activity may attenuate the rate of functional decline in young adults, highlighting the potential of lifestyle interventions to influence disease trajectories [9,10]. The identification of early decrements in lung function is clinically significant given the strong predictive value of initial impairment for future respiratory morbidity, as reflected in dose–response relationships established through indices such as the Brinkman index and peak expiratory flow [7]. These accumulating findings underscore the broader implications of tobacco use in medical students, not only for their own long-term health but also for their credibility and efficacy as future health professionals responsible for counseling patients and promoting tobacco cessation.

## 2. Materials and Methods

The increasing prevalence of chronic respiratory diseases and the impact of lifestyle factors—primarily smoking and physical activity—on lung function are receiving increasing attention in both prevention and clinical practice. This study aims to examine the effects of smoking habits, smoking duration (pack-years), and regular physical activity on the development of respiratory function parameters. The study uses quantitative data processing to explore the following specific relationships:

- Comparison of FEV_1_ values (forced expiratory volume in one second) between smoker medical students and non-smokers, demonstrating statistically significant differences between them.
- Evaluation of the effect of regular physical activity among medical students, assuming that the respiratory function values of smokers who are active athletes are significantly better than those of inactive individuals.
- Examination of physical inactivity as an independent risk factor in the decline of vital capacity, based on the assumption that a sedentary lifestyle, regardless of smoking status, can negatively affect lung compliance and ventilation efficiency
- Mapping the correlation between years spent smoking (pack/year) and lung function decline to quantitatively substantiate the exposure-damage relationship
- Exploring the extent to which active exercise can contribute to maintaining vital capacity, strengthening respiratory muscles, and reducing oxidative stress and inflammatory processes, thereby partially offsetting the harmful effects of smoking
- To compare the degree of respiratory function decline between active and inactive smoker medical students, and whether regular physical activity can mitigate the functional damage caused by smoking

The objectives of this study contribute to a better understanding of the primary behavioral factors influencing lung function and may facilitate the development of targeted prevention and education strategies, particularly among young adults.

### 2.1. Study Design and Participants

The study was a cross-sectional, observational, comparative study. The medical students included in this study volunteered to participate after attending our tobacco-related educational lectures, which provided them with relevant and comprehensive information. More than 350 students expressed interest in taking part; however, individuals with confirmed allergic asthma or allergic rhinitis, those who had experienced a viral or bacterial respiratory infection within the previous 5–10 days, as well as pregnant participants, were excluded from the study. A total of 264 medical students (101 men and 163 women) from the University of Medicine, Pharmacy, Science and Technology of Târgu-Mures, aged 18–30 (median age: 20), were included in the study. Because the tobacco-education lectures could be attended independently of academic year, medical students from the first to the fifth year were eligible to participate, if they did not meet any exclusion criteria.

Participants were categorized based on smoking status (smokers vs. non-smokers) and lifestyle habits (physically active vs. sedentary). Within the smoker’s group, we distinguished three subgroups: those who exclusively smoked traditional cigarettes, those who exclusively used electronic cigarettes or heated tobacco products, and dual users. We expressed the degree of smoking exposure using a pack/year index.

Among the medical students who reported active smoking, the typical age of smoking initiation ranged between 14 and 16 years. Pack-years index was calculated using the following standard formula: (number of cigarettes smoked per day ÷ 20) × years of smoking. During the validation of the pack-year data, we confirmed that no data entry errors or unit inconsistencies were present; the computation of this variable was performed using a uniform methodology and demonstrated internal consistency.

In order to contextualize the potential respiratory effects of electronic cigarette use, information regarding patterns of use, including device type and self-reported frequency of consumption, was collected from participants. A marked post-pandemic increase in electronic cigarette uptake was observed among adolescents and young adults, with the majority of participants reporting daily use of disposable devices (e.g., Elf Bar) or refillable pod-based systems. However, due to substantial intra-individual variability in consumption behaviors, heterogeneity in device characteristics, and limitations in recall accuracy regarding frequency and intensity of use, reliable quantitative estimates of exposure could not be consistently established across the study population. Consequently, no formal quantitative statistical analysis of usage patterns was performed, in order to avoid misclassification and overinterpretation of imprecise self-reported data.

Instead, qualitative descriptors of exposure were used to provide contextual background for the physiological measurements. This approach was further supported by existing evidence demonstrating that aerosols generated by disposable and pod-based electronic cigarettes contain substantial concentrations of propylene glycol, vegetable glycerin, nicotine salts, and multiple flavoring agents, including menthol, vanillin derivatives, ethyl maltol, benzaldehyde, and cinnamaldehyde. Several of these constituents have been shown to exert cytotoxic, pro-inflammatory, and ciliotoxic effects, particularly at the level of the peripheral airways. Consideration of these known toxicological properties allowed for an informed interpretation of the spirometric findings, despite the absence of precise quantitative exposure metrics.

Participants classified as athletes were identified using a self-reported questionnaire. Individuals were considered athletes if they engaged in physical exercise at least three times per week for a minimum of 45–60 min per session at moderate or high intensity, or if their total weekly training time reached at least 150 min of moderate-intensity activity or 75 min of high-intensity activity. Most athletic medical students participated in recreational forms of exercise, including running, swimming, tennis, and strength training. Among competitive athletes, only a small number—exclusively swimmers—took part in the study.

### 2.2. Spirometry Methods and Diagnostic Thresholds

Spirometry was performed using the SPIROLAB Spirometer with 7″ Touchscreen and SpO_2_ option (model 33520), equipped with a Miniflowmeter and a bi-directional digital turbine (flow range ± 16 L/s; accuracy ± 5% or 200 mL/s; volume accuracy ± 2.5% or 50 mL; dynamic resistance < 0.5 cmH_2_O/L/s). Data were processed using WinspiroPRO/MIR software, applying GLI-2012 reference equations with automated Z-scores and LLN calculations.

All tests met ATS/ERS acceptability and repeatability standards, including ≥3 acceptable maneuvers and reproducibility within <150 mL for FEV_1_ and FVC. Airflow obstruction was defined as FEV_1_/FVC < LLN (Z-score < −1.645). Small airway dysfunction was assessed using FEF_25–75_ < LLN and FEV_3_/FVC < LLN, based on GLI-derived thresholds.

### 2.3. Data Collection and Spirometry Testing

Standardized spirometry was performed on all participants after a 10 min rest period. The test was performed in a sitting or standing position, using a nose clip, a calibrated spirometer, and a disposable mouthpiece. The test subjects were first instructed to take several calm breaths, then to inhale as deeply as possible and exhale forcefully. The measurement was repeated three times, and the highest value was recorded.

After the functional test, we determined and analyzed the following dynamic volumes:

- Forced vital capacity (FVC)
- Forced expiratory volume in 1 s (FEV_1_)
- FEV_1_/FVC ratio (Tiffeneau index)
- Peak expiratory flow (PEF)
- Forced expiratory flow values at 75%, 50% and 25% FVC (MEF_75_, MEF_50_, MEF_25_)

#### Variables Examined and Grouping

Comparisons were made according to the following criteria:

1. Smoking status: smokers and non-smokers.
2. Lifestyle: athletes and sedentary individuals.
3. Combined subgroups: medical students who are athletes and smokers, and smoking sedentary individuals.
4. Type of tobacco products: traditional cigarettes and e-cigarettes or heated tobacco products.

### 2.4. Statistical Analysis

Data were processed using SPSS 10.13 (IBM Corp., Armonk, NY, USA). Continuous variables were presented as mean ± standard deviation or median (interquartile range, IQR), depending on their distribution. Categorical variables were described in terms of absolute numbers and percentages.

- To examine differences between groups, we used independent samples *t*-tests or Mann–Whitney U tests, and for categorical variables, we used chi-square tests.
- The correlations between smoking exposure (pack-years), physical activity, and lung function parameters were analyzed using Pearson’s correlation coefficient (r) and coefficient of determination (r^2^).
- The threshold for statistical significance was set at *p* < 0.05.

Multivariable linear regression models were applied to evaluate the association between smoking exposure and continuous spirometric outcomes (including FEV_1_, FVC, FEV_1_/FVC, and small-airway flow indices), with age and sex included as covariates. For dichotomous outcomes reflecting the presence or absence of airflow obstruction, multivariable logistic regression models were employed. All statistical analyses were performed using IBM SPSS Statistics (version 10.13), with regression coefficients, odds ratios, and corresponding 95% confidence intervals reported as appropriate.

## 3. Discussion

A total of 264 medical students from the University of Medicine, Pharmacy, Science and Technology of Târgu Mureș underwent standardized spirometry assessments of respiratory function, comprising 101 men and 163 women. We measured the lung capacity of medical students aged 18–30, with a median age of 20 years (*p* = 0.655). We found no significant difference in gender distribution, although more women than men were non-smokers (65.0% vs. 58.2%; *p* = 0.258). Spirometry was performed after adequate preparation. After 10 min of resting breathing, the subjects performed 4–5 deep inhalations and exhalations. The test was conducted in a sitting or standing position with the nose pinched. A disposable mouthpiece was placed on the spirometer, through which the subjects breathed in and out as instructed by the doctor. Participants were asked to take full inspiration and then exhale steadily into a mercury manometer via a rubber tube, maintaining a constant pressure of 40 mmHg. To meet the requirement, the individual had to retain the mercury column at this level for at least 6 s.

After a few quiet breaths, we asked the study participant to take a forced maximum inhalation, followed by a forced maximum exhalation. The forced expiratory volume (FVC) was measured three times, and the highest value was recorded. The subjects were prohibited from smoking or engaging in strenuous physical activity for at least 1 h before the test. This allowed us to rule out any negative respiratory function results caused by potential risk factors. After the functional test, we determined and analyzed the dynamic volumes. Of the time-dependent volumes, we placed particular emphasis on forced vital capacity (FVC), forced expiratory volume in one second (FEV_1_), the Tiffeneau index (FEV_1_/FVC), and forced expiratory vital capacity at 75%, 50% and 25% flow rates (MEF_75_, MEF_50_, MEF_25_).

As detailed in Table 1, the study cohort is stratified into two primary categories. We distinguished between smokers and non-smokers, as well as between athletes and medical students with a sedentary lifestyle. Of the 264 medical students examined, 124 confirmed that they were smokers, while 140 were classified as non-smokers. We classified smokers into three main categories. We observed the differences in respiratory function parameters between classic smokers and the group of electronic cigarette (E-cigarettes, IQOS) users, and we analyzed separately all individuals who could be classified into both categories. Of the 124 smokers, 71 women (57.3%) and 53 men (42.7%) participated in the study. Of the smokers, 102 individuals (82.258%) were classified as classic cigarette consumers, while 57 individuals (47.581%) were classified as users of electronic cigarettes or heated tobacco products (IQOS). Among medical students who smoke, regardless of cigarette type, the median pack-year value was 4 (IQR: 4–5), while among non-smokers it was 0 (*p* < 0.001).

A key objective was to determine whether repeated weekly engagement in physical activity can augment lung compliance in smokers, thereby counteracting, at least in part, the deleterious physiological consequences of tobacco exposure. In contrast, we hypothesized that all subjects who smoke and lead a sedentary lifestyle would have poorer respiratory function than those who smoke but also engage in sports several times a week, which has a positive effect on the respiratory muscles. Based on our results, we demonstrated that regular sports activities were significantly more common among non-smokers (67.1%) than among smokers (45.2%; *p* < 0.001). The distribution of sports participation frequency across the two groups further supported the protective association of an active lifestyle with respiratory health. Numerous foreign studies have similarly demonstrated the positive impact of various sports activities on lifestyle. A survey conducted in the United States, which examined the role of physical activity in reducing the links between smoking, sleep disorders, and symptoms of depression among the adult population, stated that although current evidence is limited and partly contradictory, regular participation in moderate-intensity physical activity may mitigate some of the harmful effects of smoking, particularly those related to cardiovascular health and reduced lung capacity [13]. At the same time, our main objective was to prove that smoking causes significant, objectively measurable damage to respiratory function parameters even at a young age and increases the risk of developing obstructive lung diseases. Based on our results, we can conclude that although the study population is young, smoking already has a demonstrable detrimental effect on respiratory function at this age. These results underscore the importance of early prevention programs, particularly among young adults and medical university students, to prevent the decline in lung function.

### Comparison of Medical Student Athletes Who Smoke and Smokers Who Lead a Sedentary Lifestyle

Comparative analyses were undertaken to examine differences in lung function between smoking medical students who engaged in regular physical activity (n = 56) and those who remained physically inactive. The results showed significant differences across all variables examined, confirming a significantly more favorable effect of regular physical activity on respiratory function. A comparable perspective was provided by a cross-sectional study conducted among the elderly population in India, which examined variations in respiratory function parameters, particularly maximal expiratory flow, between physically active individuals and their sedentary counterparts. The findings of this investigation highlight the crucial role of regular physical activity, particularly aerobic forms such as walking and swimming, in strengthening respiratory muscles and improving endurance. Moreover, the analysis confirmed that an active lifestyle exerted a protective association, mitigating the age-related decline in pulmonary function [14].

Based on Table 2, we can conclude that the median value of peak expiratory flow (PEF) (median: 95.0, IQR: 80.0–110.5) was significantly higher than that of medical students characterized by physical inactivity (median: 63.5, IQR: 56.0–76.0; *p* < 0.001), reflecting better airway patency and more efficient ventilation performance. A similar trend was observed for forced expiratory volume in one second (FEV_1_), where there was a significant difference between the median value for medical student athletes (106.5, IQR: 99.0–114.0) and physically inactive medical students (88.0, IQR: 82.75–95.0, *p* < 0.001). The results for forced vital capacity (FVC) also showed a significant difference, with higher averages for medical student athletes (median: 109.64 ± 11.898), suggesting a more favorable preservation of lung function. The FEV_1_/FVC ratio (Tiffeneau index) of athlete students was significantly more favorable (83.60) than that of medical students with a sedentary lifestyle (78.75; *p* < 0.001), suggesting that ventilation disorders are less common in those with an active lifestyle. Numerous scientific studies have demonstrated that regular physical activity protects against the development of chronic diseases and reduces the risk of premature mortality. A fascinating Canadian study, which targeted people aged 45–85, analyzed healthy individuals, those with asthma, and those with very low FEV_1_ values, and concluded that, in both healthy individuals and those with obstructive diseases, FEV_1_ and FVC indices showed a positive correlation with physical activity. Thirty minutes of strength training or intense endurance training per day resulted in a 0.65 percentage point increase in the FEV_1_ index, while the FVC increased by an average of 0.49 percentage points. These results suggest that physical activity may be beneficial for both healthy individuals and those with respiratory diseases [15,16]. In two population-based cohort studies involving children and young adults, Hancox et al. demonstrated that aerobic capacity is closely and positively correlated with FEV_1_ and FVC values measured in young populations. However, they were unable to confirm a correlation between the FEV_1_/FVC ratio and age, suggesting that aerobic performance is more closely related to lung volume than to airway diameter. Based on their findings, it can be inferred that the development of aerobic capacity in childhood and adolescence may have a beneficial effect on lung function in later life [17].

**Figure 1:**
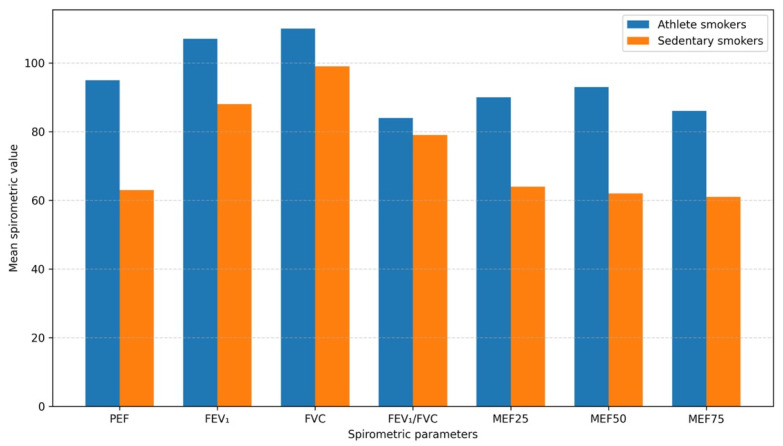
Comparison of lung function parameters between medical student athletes who smoke and sedentary smokers. PEF: Peak expiratory flow; FEV_1_: Forced Expiratory Volume in 1 s; FVC: Forced Vital Capacity; MEF_25_ Maximal Expiratory Flow at 25%; MEF_50_: Maximal Expiratory Flow at 50%; MEF_75_: Maximal Expiratory Flow at 75%.

In terms of flow parameters characteristic of the small airways (MEF_25_, MEF_50_, MEF_75_), students with an active lifestyle also showed a clear advantage (89.8%, 92.5%, 86.0%), suggesting that regular physical activity has a beneficial effect on reducing airway resistance and preserving peripheral airway function. The incidence of airway obstruction showed a particularly striking difference between the two groups studied. At the level of the large airways, obstruction was identified in 25% of physically inactive medical students (*n* = 17), whereas it did not occur in any of the medical student athletes (*p* < 0.001). At the level of the small airways, peripheral dysfunction was detected in 21.4% of student athletes (*n* = 12) and in 82.4% of medical students characterized by physical inactivity (*n* = 56) (*p* < 0.001). Overall, the data obtained (Figure 1) clearly indicates that although both groups studied consisted of smokers, regular physical activity can significantly moderate the decline in lung function, reduce airway inflammation, and decrease the incidence of associated obstruction even in a smoking population. Moreover, the case–control analysis demonstrated that an active lifestyle was independently associated with a protective effect, attenuating the age-related decline in pulmonary function. Numerous systematic reviews have found that comprehensive treatment and rehabilitation programs developed for individuals with chronic obstructive pulmonary disease emphasize the recognized key role of physical activity in enhancing patient well-being, managing symptoms, and improving overall quality of life. A foreign systematic review, which assessed intervention studies that evaluated physical activity as an outcome measure in COPD patients, suggested that individuals with obstructive disease should monitor their daily and weekly physical activity and use objective measurement tools to track a minimum walking exercise program of at least 30 min per day, at least 3 times per week. At the same time, Kantorowski et al. proposed a system based on a behavior self-regulation theory, a pedometer, and a website to increase patients’ activity levels, allowing the COPD population to set goals, receive feedback, and access motivational and educational tips. These motivational techniques have been shown to increase daily step counts and improve lung capacity parameters [18,19].

One of the primary objectives of our study was to examine the impact of classic cigarette consumption, smoking exposure (packs/year), and participation in sports activities on various respiratory function parameters in medical students from the University of Medicine, Pharmacy, Science, and Technology of Târgu-Mureș. During the analyses, we used Pearson’s correlation coefficient (r), coefficient of determination (r^2^), and *p*-values to evaluate the significance of the correlations. The results showed clear and statistically significant correlations between the lifestyle factors examined and respiratory function (Table 3).

When comparing the data presented in Table 3, we observed particularly pronounced differences between smokers and non-smokers in the MEF_25_, MEF_50_, and MEF_75_ values, which reflect the function of the small airways. These parameters demonstrated a negative correlation with smoking, as tobacco use, irrespective of type, increased the likelihood of developing mild to moderate narrowing at the level of the distal airways. Our findings align with previous reports that the small airways are particularly susceptible to tobacco smoke-induced inflammatory and structural changes, resulting in increased mucus secretion, smooth muscle hypertrophy, and lumen narrowing, which may serve as an early indicator of functional airway narrowing [20,21]. Early indicators of peripheral narrowing caused by smoking are precisely reflected in the decrease in flow parameters. A foreign meta-analysis has highlighted that the MEF_75_ value may be the most sensitive indicator for confirming increased exposure to cigarette smoke, and its decrease may be a primary marker for obstructive disease at the level of the small airways, even in individuals with FEV_1_ within the normal range. However, it is essential to note that MEF_75_ exhibits greater variability, is highly dependent on forced vital capacity, and has a wide range; therefore, it cannot replace FEV_1_ as a standalone measure, which remains the primary indicator of airway flow impairment in smokers [22].

Classic cigarette smoking showed a moderate to strong negative correlation with several respiratory function parameters. There was a strong positive correlation with distal ventilatory obstruction (OVD) (r = 0.595, r^2^ = 0.354), which supports the well-known role of smoking in the pathophysiological mechanism of chronic obstructive pulmonary disease (COPD), causing persistent inflammation and distal airway damage. Long-term exposure caused not only changes in the larger airways, but also progressive signs of involvement of the peripheral airways, emphasizing the worsening of the obstructive pattern. A similar idea was expressed in a pivotal study involving 75 asymptomatic smokers with preserved spirometry values. It was observed that reduced MEF_25–75_ parameters often occur even in the absence of flow limitation. In asymptomatic smokers, in addition to the early presence of small airway obstruction, a parallel deterioration in parameters obtained by classical spirometry was detected, indicating worsening flow limitation [23]. The correlations between pack-years/index and respiratory function parameters are particularly valuable in assessing the effects of cumulative exposure. The decrease in MEF75 values (r = −0.438, *p* < 0.001) and an increase in distal airway resistance (r = 0.575, *p* < 0.001) suggest a more pronounced adverse effect of smoking duration and quantity, which over time leads to damage to the small airways. At the same time, in the case of the Tiffeneau index (FEV_1_/FVC), it was observed that even at a relatively low r value (r = −0.41), the coefficient of determination represented an explanatory power of 15–20%, which may be of high clinical significance. These results are consistent with the recommendations of the Global Obstructive Lung Disease (GOLD) initiative, according to which smoking history is a key risk factor in predicting functional decline in the lung parenchyma [24].

In contrast, significantly better respiratory function parameters were observed among medical students engaged in athletics. Athlete status showed a strong positive correlation with PEF (r = 0.602, *p* < 0.001), FEV1 (r = 0.588, *p* < 0.001), and MEF_25_ (r = 0.598, *p* < 0.001). In addition, sports activity moderately but significantly improved the FEV1/FVC ratio (r = 0.416, *p* < 0.001) and had a favorable effect on small airway flow parameters, including MEF_50_ (r = 0.558, *p* < 0.001) and MEF_75_ (r = 0.449, *p* < 0.001). The beneficial effects of exercise have been confirmed by numerous studies, which show that regular aerobic exercise improves lung function and reduces obstructive airway symptoms, even in smokers. For example, in a foreign study, Andrade CHS et al. observed the effect of physical activity on the treatment of asthma patients. Based on their results, they demonstrated that increased physical performance improves psychosocial factors in individuals with obstructive pulmonary disease, reducing exercise-induced bronchoconstriction and the need for corticosteroid use. These results suggest that an active lifestyle may play a role in alleviating airway inflammation and associated narrowing [25]. A novel observation is that exercise is negatively correlated with the onset of obstructive ventilation disorders (r = −0.488), leading to a decrease in distal airway resistance. This may suggest that an active lifestyle can partially offset the adverse peripheral airway effects of smoking, exerting a protective effect. The correlations we found were also directly reflected in a foreign meta-analysis, where the authors, comparing several randomized controlled trials, measured the positive impact of exercise on smoking cessation and stated that regular aerobic exercise not only improves cardiovascular and respiratory function, but also alleviates obstructive airway symptoms even among smokers [26].

**Figure 2:**
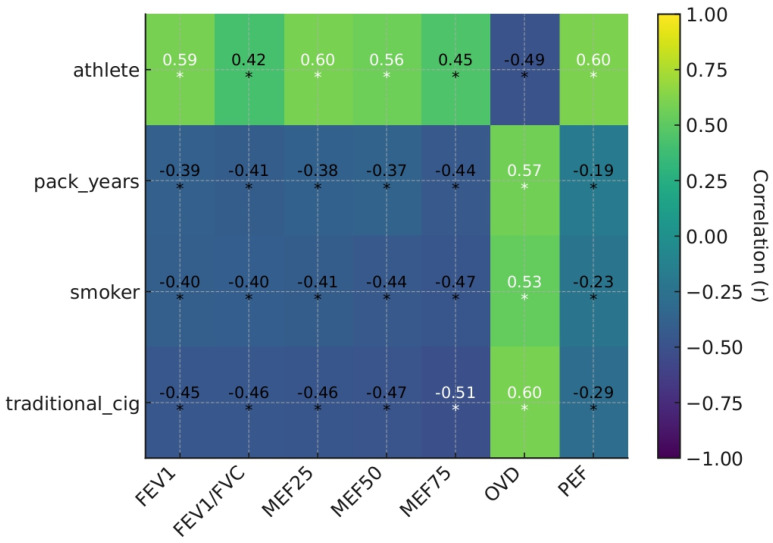
Correlation heatmap (Pearson r) across lifestyle/smoking factors and lung function parameters. PEF: Peak expiratory flow; FEV_1_: Forced Expiratory Volume in 1 s; FVC: Forced Vital Capacity; MEF_25_: Maximal Expiratory Flow at 25%; MEF_50_: Maximal Expiratory Flow at 50%; MEF_75_: Maximal Expiratory Flow at 75%; “*”: Indicates statistically significant correlations (*p* < 0.05).

Overall, as confirmed by the correlation heat map (Figure 2), respiratory function parameters show significant sensitivity to lifestyle factors. Smoking harms both large and small airway functions, and cumulative exposure further exacerbates functional decline. In contrast, regular physical activity has a beneficial effect on several parameters and can partially offset the harmful effects of lifestyle factors, compensating for the decrease in airway function. The strength and significance of the correlations confirm the clinical relevance of the data obtained, emphasizing the importance of smoking cessation and promoting physical activity from both a prevention and intervention perspective.

Peak expiratory flow (PEF) was significantly higher in the group using alternative tobacco products (88.5 vs. 73.0; *p* = 0.009), suggesting that maximum expiratory dynamics are better preserved in this student population.

The forced expiratory volume in the first second (FEV_1_) showed a particularly significant difference: in the e-cigarette/IQOS group, the mean function exceeded 100% (104.8 ± 11.3), while in traditional smokers, we recorded a significantly lower value (93.4 ± 14.9; *p* < 0.001). This trend was also reflected in the FEV_1_/FVC ratio, where the values for alternative product users were more favorable (84.6 vs. 80.7; *p* < 0.001), suggesting less obstructive abnormalities.

The flow parameters of the small airways (MEF_25_, MEF_50_, MEF_75_) were consistently higher among e-cigarette/heated tobacco product users. For example, the median MEF_25_ value was 87.5, compared to 68.0 for conventional smokers (*p* < 0.001). These differences suggest that functional impairment of the peripheral airways is less pronounced in those who use e-cigarettes or heated tobacco products. However, a randomized crossover study found that acute, 5-day use led to a 25% increase in forced expiratory flow (MEF_25_), suggesting that discontinuing the use of heated tobacco products reduced airway resistance, thereby improving lung function. Based on this, we can conclude that even short-term exposure to e-cigarette aerosol can lead to increased airway resistance and inflammation, which, however, may be reversible upon cessation of these products [27].

In our study, we focused on comparing different smoking habits and assessing the negative impact of these harmful habits on respiratory function parameters. A total of 124 medical students who are smokers participated in the study, of whom 102 smoked conventional cigarettes, while 22 used heated tobacco products or e-cigarettes (IQOS/e-cigarettes). Some students fell into both categories. During the study, we found significant differences between the respiratory function indicators of traditional smokers and e-cigarette/IQOS users (Table 4).

Peak expiratory flow (PEF) was significantly higher in the group using alternative tobacco products (88.5 vs. 73.0; *p* = 0.009), suggesting that maximum expiratory dynamics are better preserved in this population.

Forced expiratory volume in one second (FEV_1_) showed a particularly significant difference: in the e-cigarette/IQOS group, the average function exceeded 100% (104.8 ± 11.3), while in traditional smokers, we recorded a significantly lower value (93.4 ± 14.9; *p* < 0.001), predicting the possibility of developing obstructive pulmonary disease. A similar idea was expressed in a retrospective cohort study, which stated that FEV_1_ is one of the most important parameters used in the diagnosis and monitoring of smoking-related diseases. Chronic exposure to cigarette smoke causes inflammation and narrowing of the airways, which, over time, leads to a gradual decline in FEV_1_. This decline reflects airway obstruction and is associated with worsening respiratory symptoms, decreased physical performance, and an increased risk of exacerbations, confirming the clinical relevance of FEV_1_ [28]. The trend observed in FEV_1_ levels was also reflected in the FEV_1_/FVC ratio, where values were more favorable for users of alternative tobacco products (84.6 vs. 80.7; *p* < 0.001), confirming fewer obstructive abnormalities in individuals belonging to this group. The flow parameters of the small airways (MEF_25_, MEF_50_, MEF_75_) were consistently higher among users of e-cigarettes/heated tobacco products (IQOS). For example, the median MEF_25_ value was 87.5, compared to 68.0 for conventional smokers (*p* < 0.001). These differences suggest that functional impairment of the peripheral airways is less pronounced in those who use e-cigarettes or heated tobacco products. In contrast, a Greek study’s results showed that after using heated tobacco products, especially IQOS (“I-Quit-Ordinary-Smoking”), participants’ gas exchange and small airway functional parameters (SaO2, MEF_25_%, MEF_50_%, MEF_75_%, PEF) significantly deteriorated. In contrast, there was an increase in exhaled CO levels and airway resistance measured at multiple frequencies (R5-R35 HZ), indicating acute airway stress and increased obstructive changes [29]. However, a randomized crossover study found that acute, 5-day use led to a 25% increase in forced expiratory flow (MEF_25_), suggesting that discontinuing the use of heated tobacco products reduced airway resistance, thereby improving lung function. Based on this, we can conclude that even short-term exposure to e-cigarette aerosol can lead to increased airway resistance and inflammation, which, however, may be reversible upon cessation of these products [27].

Vital capacity (FVC) was also higher among e-cigarette/IQOS users (108.0 vs. 103.0). Still, the difference was not statistically significant (*p* = 0.082), which suggests that total lung capacity is a less sensitive marker of the effects of different smoking habits and draws attention to the inevitable negative impact of e-cigarette aerosol exposure on the cardiovascular system. Similarly, an American study examining the cardiorespiratory effects of heated tobacco products found that e-cigarettes, e-liquids, and the aerosols they emit contain both known and unknown harmful chemicals that can cause increased arterial stiffness, vascular endothelial changes, and heightened airway reactivity and inflammation, similar to traditional tobacco products. Although e-cigarettes are advertised as a healthier alternative to conventional cigarettes, research findings to date show that the respiratory and cardiovascular systems undergo numerous changes and that the development of disease depends on how these changes combine with environmental and genetic factors [30]. However, a recent study comparing smokers without chronic disease to a group of asthma patients treated with short-acting bronchodilators stated that exposure to a single electronic cigarette or heated tobacco product can cause immediate mechanical and respiratory changes in both study groups, with the intensity and duration of the changes being more pronounced in individuals with asthma symptoms [31].

Overall, based on our test results, we can conclude that although both forms of smoking can cause damage to the respiratory system, respiratory function parameters were more favorable in several respects in users of e-cigarettes/heated tobacco products (IQOS). The most significant differences were observed in small airway flow values, as well as in FEV_1_ and the FEV_1_/FVC ratio, which clinically suggests that traditional smoking leads to faster and more severe respiratory function decline. Although alternative devices cannot be considered harmless, these results may support the idea that switching from conventional smoking may have certain physiological benefits, potentially leading to a reduction in the levels of pulmonary endothelial and inflammatory markers. Further prospective, long-term studies are needed to determine clinical relevance, more accurately assess the extent of risk reduction, and evaluate the potential for the development of obstructive lung diseases. Our findings revealed an inverse correlation compared with previously published reports, which concluded that heated tobacco products (HTPs), including IQOS, exhibit considerable toxicity and may trigger pathophysiological mechanisms like those induced by conventional cigarettes [32]. In a 2024 study examining the effects of switching from traditional cigarettes to IQOS on pulmonary endothelial and inflammatory biomarkers, the authors reported that transitioning to heated tobacco products resulted in only modest improvements. In contrast, complete cessation was substantially more effective in restoring both immune function and pulmonary performance [33]. Furthermore, an independent investigation conducted in 2018 among otherwise healthy adults—who reported smoking at least 10 conventional cigarettes per day over the preceding three years—demonstrated no significant differences in respiratory parameters between IQOS users and conventional smokers, thereby reinforcing evidence that HTPs exert detrimental effects by promoting airway inflammation and immunosuppression [34].

Finally, but not least, we also compared the obtained lung function parameters between the group of medical student athletes and those with a sedentary lifestyle. The results presented in Table 5 clearly demonstrate that athletically active medical students exhibited significantly more favorable lung function values compared with their sedentary counterparts. These differences were evident across a wide range of parameters, supporting the beneficial impact of regular physical activity on respiratory function and capacity. The peak expiratory flow (PEF) and forced expiratory volume (FEV_1_) values were significantly higher in the group of student athletes, indicating improved expiratory performance and more efficient airway functioning. A similar trend can be observed in the case of vital capacity (FVC), where physically active medical students demonstrated a statistically significant difference in lung capacity compared to those with a physically inactive lifestyle. The FEV_1_/FVC ratio also confirmed the more favorable respiratory function profile of student athletes, suggesting that sports activities contribute to maintaining airway elasticity and expiratory dynamics. A 2023 study examining anthropometric parameters in young athletes compared with individuals leading a sedentary lifestyle reported broad agreement regarding the beneficial role of regular aerobic exercise in reducing the burden of respiratory diseases. However, the direct effect of an active lifestyle on mortality and disease burden reduction remains inconclusive. The investigation nonetheless identified apparent differences in pulmonary function indices between athletes and non-athletes, with significantly higher PEF and FEV_1_ values observed in the athletic cohort, thereby underscoring the respiratory benefits of sustained physical activity [35]. Similarly, a 2024 descriptive study assessing pulmonary function parameters in individuals aged 18 to 80 years demonstrated that increased sedentary behavior is negatively associated with lung function indices and can be identified as a significant risk factor for the development of several chronic diseases, including respiratory disorders [36].

Notably, the consistently higher values of small airway flow parameters (MEF_25_, MEF_50_, MEF_75_) among individuals participating in competitive sports indicate better peripheral airway patency and more efficient airflow maintenance. In a descriptive and comparative cross-sectional study conducted over six months in 2023, in which sedentary individuals were compared with elite athletes, it was reported that elite athletes may also develop maladaptive alterations at the level of the respiratory system, such as intra- and extra-thoracic obstructions, expiratory flow limitation, respiratory muscle fatigue, and exercise-induced hypoxemia. Nevertheless, the study demonstrated that athletes exhibit superior airway patency and more favorable small-airway flow parameters (MEF_25–75_). The reduced airway resistance may partly explain the findings of increased alveolar compliance and improved overall lung elasticity observed in elite athletes [37].

Overall, these results confirm that regular physical activity has a beneficial effect not only on the cardiovascular system but also on respiratory function. The significant differences observed in student athletes reflect multidimensional improvements in lung mechanics and airway function. Accordingly, the findings support the conclusion that regular exercise functions as a protective factor against the decline in respiratory function and contributes substantially to the maintenance of long-term respiratory health.

To obtain a more refined and robust understanding of the detrimental effects of smoking and the beneficial impact of physical activity, we conducted a multivariable regression analysis adjusted for sex and age. This approach allowed us to account for key demographic confounders and thereby strengthen the validity and interpretability of our findings (Table 6).

Across all multivariable models, the influence of smoking-related variables on ventilatory function was generally limited, whereas demographic and lifestyle predictors demonstrated more consistent and clinically meaningful associations. For the PEF (% predicted) model (R^2^ = 0.436), none of the smoking-related variables showed significant independent effects, including smoking status, pack-years, classical cigarettes, or electronic/heat-not-burn devices. Only age, male sex, and particularly regular sports activity emerged as significant positive predictors, underscoring physiological growth effects and the beneficial influence of cardiorespiratory conditioning (Table 7).

This pattern was also evident in the regression model for FEV1/FVC (R^2^ = 0.365), where classical smoking indicators, including smoking status and pack-years, did not emerge as significant determinants of expiratory flow (Table 8). Although some trends were observable (e.g., pack-years approaching significance for FEV1/FVC), these effects remained modest and imprecise, suggesting that early smoking exposure in this predominantly young population has not yet translated into measurable impairments in flow ratios (Table 8).

Similarly, in the multivariable linear regression model for FEV1% (R^2^ = 0.459), most smoking-related predictors did not demonstrate significant independent associations. Neither current smoking status nor the use of conventional cigarettes showed meaningful effects on FEV1%, and pack-years displayed only a borderline negative trend. Electronic cigarette/IQOS use showed a modest positive association, though this should be interpreted with caution given the potential for behavioral or selection-related bias. Age and sex were again not significant predictors. Regular physical activity emerged as the strongest determinant of FEV1%, being independently associated with substantially higher values, underscoring the prominent role of lifestyle factors in preserving ventilatory capacity within this cohort (Table 9).

This pattern was also evident in the regression models for MEF25 (R^2^ = 0.483), MEF50 (R^2^ = 0.454), and MEF75 (R^2^ = 0.382), where classical smoking indicators, including smoking status and pack-years, did not emerge as significant determinants of expiratory flow (Table 10, Table 11 and Table 12). Although some trends were observable (e.g., pack-years approaching significance for MEF75), these effects remained modest and imprecise, suggesting that early smoking exposure in this predominantly young population has not yet translated into measurable impairments in flow–volume curve segments (Table 12).

In contrast, as shown in Table 10 and Table 11, electronic cigarette or heated tobacco use showed small but statistically significant positive associations in several models (MEF_25_ and MEF_50_). While statistically detectable, these effects are unlikely to represent physiological improvement; they may instead reflect behavioral patterns, differences in inhalation technique, or residual confounding. Such findings should therefore be interpreted with caution.

Age exerted significant positive effects on MEF_25_ but did not influence the other flow parameters or ratios, likely reflecting the relatively narrow age distribution of the cohort. Being male was associated with lower FEV_1_/FVC values and showed borderline effects in other models, a finding consistent with known sex-related differences in airway geometry and spirometry performance. Across all models, engaging in regular sports activity was among the strongest predictors of improved expiratory flow, demonstrating robust positive effects on MEF_25_, MEF_50_, MEF_75_, and, to a lesser extent, FEV_1_/FVC. These results underscore the protective influence of physical conditioning and respiratory muscle performance, which may partially counterbalance early adverse exposures (Table 8, Table 10, Table 11 and Table 12).

As shown in Table 6 and Table 13, the logistic regression models consistently identified cumulative smoking exposure (pack-years) as the only significant smoking-related predictor of distal airway obstruction, demonstrating a clear dose–response relationship. Current smoking, classical cigarette use, and electronic cigarette/heat-not-burn use showed non-significant associations, indicating no measurable independent effect in this cohort. Age and sex were likewise non-significant predictors. In contrast, regular sports participation emerged as a strong and consistent protective factor, markedly reducing the odds of distal airflow obstruction. Overall, the combined findings indicate that cumulative smoke exposure is the key determinant of early distal airway involvement, while sustained physical activity provides substantial protection. For proximal obstruction (large-airway DVO ≥ 1), the pattern was similar, with minimal influence from smoking-related variables and a protective trend associated with sports participation. These findings reinforce the concept that in young individuals, airflow limitation, when present, is more closely linked to cumulative exposure rather than smoking status alone, and that physical activity plays a central role in preserving airway function.

For proximal obstruction (large-airway DVO ≥ 1), the pattern was similar, with minimal influence from smoking-related variables and a protective trend associated with sports participation. These findings reinforce the concept that in young individuals, airflow limitation, when present, is more closely linked to cumulative exposure rather than smoking status alone, and that physical activity plays a central role in preserving airway function.

Overall, the collective results suggest that demographic and lifestyle factors currently outweigh smoking-related predictors in determining spirometry performance within this cohort. The early and subtle trends associated with cumulative smoking exposure highlight the potential for progression, emphasizing the importance of longitudinal follow-up. The consistently strong beneficial effects of regular sports activity across all outcomes underscore its relevance as a modifiable determinant of respiratory health, even among individuals with early exposure to tobacco or nicotine products.

## 4. Conclusions

The results demonstrate that smoking is associated with significant reductions in vital capacity, forced expiratory volume, and small-airway flow, indicating early and progressive impairment of respiratory function. Although users of e-cigarettes and heated tobacco products exhibited comparatively more favorable spirometry values than conventional cigarette smokers, these alternative products still generate aerosols containing toxic compounds linked to vascular and airway injury and therefore cannot be considered harmless. Regular physical activity was associated with consistently more favorable respiratory parameters, suggesting a partial protective effect against smoking-related functional decline. Overall, these findings underscore the importance of integrated prevention strategies that combine tobacco control measures with physical activity promotion to preserve lung function and reduce respiratory morbidity.

## Figures and Tables

**Table 1:** Comparison of respiratory function values between medical student smokers and non-smokers.

Variable	Smoker (*n* = 124)	Non-Smoker (*n* = 140)	*p*-Value
Age	20.00 (19.00–22.00)	20.00 (19.00–23.00)	0.655
Female	71% (58.2%)	91% (65.0%)	0.258
Conventional cigarette	102 (82.3%)	0 (0.0%)	<0.001
E-cigarette/IQOS	59 (47.6%)	0 (0.0%)	<0.001
Pack-years	4.00 years (4.00–5000)	0.00 years (0.00–0.00)	<0.001
Athlete	56% (45.2%)	94 (67.1%)	<0.001
PEF	76.50% (61.50–98.25%)	89.00% (75.75–98.50%)	<0.001
FEV1	96.50% (87.00–106.00%)	105.00% (98.00–115.00%)	<0.001
FVC	104.00% (96.00–112.50%)	107.00% (98.00–117.00%)	0.030
FEV1/FVC	81.200% (75.45–85.20%)	85.90% (81.70–90.72%)	<0.001
MEF25	73.00% (57.75–92.00%)	93.00% (82.75–103.25%)	<0.001
MEF50	75.50% (59.75–93.00%)	98.00% (82.75–114.00%)	<0.001
MEF75	68.00% (55.00–87.00%)	98.50% (84.00–119.25%)	<0.001
OVD large airways	17% (13.7%)	0% (0.0%)	<0.001
OVD small airways	68% (54.8%)	8% (5.7%)	<0.001

PEF: Peak expiratory flow; FEV1: Forced Expiratory Volume in 1 s; FVC: Forced Vital Capacity; MEF_25_: Maximal Expiratory Flow at 25%; MEF_50_: Maximal Expiratory Flow at 50%; MEF_75_: Maximal Expiratory Flow at 75%; OVD: Obstructive Ventilatory Dysfunction.

**Table 2:** Comparison of respiratory function values between medical student athletes and sedentary individuals.

Variable	Athlete (*n* = 56)	Sedentary (*n* = 68)	Test	*p*-Value
PEF	95.00% (80.00–110.50%)	63.50% (56.00–76.00%)	Mann–Whitney U test	<0.001
FEV_1_	106.50% (99.00–114.00%)	88.00% (82.75–95.00%)	Mann–Whitney U test	<0.001
FVC	109.63 ± 11.89%	98.10 ± 13.83%	*t*-test	<0.001
FEV1/FVC	83.60% (79.07–87.60%)	78.75% (70.07–82.37%)	Mann–Whitney U test	<0.001
MEF25	89.80 ± 20.13%	63.89 ± 16.04%	*t*-test	<0.001
MEF50	92.50% (78.00–105.00%)	62.00% (54.00–75.50%)	Mann–Whitney U	<0.001
MEF75	86.00% (68.75–99.25%)	61.00% (47.500–69.25%)	Mann–Whitney U test	<0.001
Obstruction in large airways	0% (0.00%)	17% (25.00%)	Chi-square test	<0.001
Obstruction in small airways	12% (21.42%)	56% (82.35%)	Chi-square test	<0.001

PEF: Peak expiratory flow; FEV1: Forced Expiratory Volume in 1 s; FVC: Forced Vital Capacity; MEF_25_: Maximal Expiratory Flow at 25%; MEF_50_: Maximal Expiratory Flow at 50%; MEF_75_ Maximal Expiratory Flow at 75%.

**Table 3:** The association between lifestyle variables and lung function parameters.

Variable 1	Variable 2	r	r^2^	*p*-Value
Smokers/Non-smokers	MEF25	−0.411	0.169	<0.001
Smokers/Non-smokers	MEF50	−0.444	0.197	<0.001
Smokers/Non-smokers	MEF75	−0.468	0.219	<0.001
Smokers/Non-smokers	OVD	0.525	0.276	<0.001
Traditional cigarette	FEV1	−0.454	0.206	<0.001
Traditional cigarette	FEV1/FVC	−0.461	0.212	<0.001
Traditional cigarette	MEF25	−0.462	0.214	<0.001
Traditional cigarette	MEF50	−0.472	0.222	<0.001
Traditional cigarette	MEF75	−0.510	0.260	<0.001
Traditional cigarette	OVD	0.595	0.354	<0.001
Pack-years	FEV1/FVC	−0.410	0.168	<0.001
Pack-years	MEF75	−0.438	0.192	<0.001
Pack-years	OVD	0.575	0.330	<0.001
Athlete: Yes/No	PEF	0.602	0.362	<0.001
Athlete: Yes/No	FEV1	0.588	0.346	<0.001
Athlete: Yes/No	FEV1/FVC	0.416	0.173	<0.001
Athlete: Yes/No	MEF25	0.598	0.357	<0.001
Athlete: Yes/No	MEF50	0.558	0.311	<0.001
Athlete: Yes/No	MEF75	0.449	0.202	<0.001
Athlete: Yes/No	OVD	−0.488	0.238	<0.001

PEF: Peak expiratory flow; FEV_1_: Forced Expiratory Volume in 1 s; FVC: Forced Vital Capacity; MEF_25_: Maximal Expiratory Flow at 25%; MEF_50_: Maximal Expiratory Flow at 50%; MEF_75_: Maximal Expiratory Flow at 75%; OVD: Obstructive Ventilatory Dysfunction.

**Table 4:** Comparison of medical student smokers who smoke traditional cigarettes and e-cigarette/IQOS users.

Parameter	Traditional Smoker (*n* = 102)	E-Cigarette/IQOS User (*n* = 22)	*p*-Value
PEF	73.00% (58.25–98.50%)	88.50% (81.00–97.75%)	0.009
FEV1	93.44 ± 1.91%	104.81 ± 11.26%	<0.001
FVC	103.00% (93.00–111.75%)	108.00% (102.50–115.50%)	0.082
FEV1/FVC	80.70% (72.60–83.97%)	84.55% (81.85–89.62%)	<0.001
MEF25	68.00% (57.00–87.00%)	87.50% (75.75–96.75%)	<0.001
MEF50	68.00% (57.00–89.75%)	90.00% (78.00–98.75%)	0.003
MEF75	66.00% (52.00–82.25%)	87.50% (68.25–100.75%)	<0.001

PEF: Peak expiratory flow; FEV_1_: Forced Expiratory Volume in 1 s; FVC: Forced Vital Capacity; MEF_25_: Maximal Expiratory Flow at 25%; MEF_50_: Maximal Expiratory Flow at 50%; MEF_75_: Maximal Expiratory Flow at 75%.

**Table 5:** Comparison of lung function parameters in non-smoking medical student athletes and sedentary individuals.

Parameter	Athlete (*n* = 94)	Sedentary Lifestyle (*n* = 46)	Test	*p*-Value
PEF	96.02 ± 15.32%	75.26 ± 12.44%	*t*-test	<0.001
FEV1	111.21 ± 11.55%	98.67 ± 8.54%	*t*-test	<0.001
FVC	111.00% (99.25–119.50%)	100.50% (95.50–112.75%)	Mann–Whitney U test	0.004
FEV1/FVC	87.47 ± 6.04%	83.43 ± 5.82%	*t*-test	<0.001
MEF25	98.80 ± 12.95%	80.13 ± 13.04%	*t*-test	<0.001
MEF50	105.03 ± 17.54%	86.04 ± 16.58%	*t*-test	<0.001
MEF75	101.00% (89.00–123.75%)	86.00% (70.25–107.75%)	Mann–Whitney U test	<0.001

PEF: Peak expiratory flow; FEV_1_: Forced Expiratory Volume in 1 s; FVC: Forced Vital Capacity; MEF_25_: Maximal Expiratory Flow at 25%; MEF_50_: Maximal Expiratory Flow at 50%; MEF_75_: Maximal Expiratory Flow at 75%.

**Table 6:** Multivariable logistic regression analysis of airway obstruction by Sex and Age.

Outcome	Predictor	OR	95% CI	*p*
Distal obstruction (any vs. none)	Sex (male vs. female)	0.85	0.49 to 1.49	0.58
Distal obstruction (any vs. none)	Age (per 1 year)	0.98	0.87 to 1.10	0.82
Proximal obstruction (any vs. none)	Sex (male vs. female)	1.50	0.55 to 4.07	0.42
Proximal obstruction (any vs. none)	Age (per 1 year)	0.96	0.78 to 1.20	0.77

**Table 7:** Multivariable linear regression models with adjustment for PEF (% of predicted) R^2^ = 0.436.

Independent Variable	Beta	95% CI Low	95% CI High	*p*
Smoker/Non-smoker	−0.93	−10.58	8.71	0.84
Pack years	−0.06	−1.53	1.40	0.93
Traditional cigarette	−6.22	−14.79	2.35	0.15
E-cigarette/IQOS	2.48	−3.77	8.75	0.43
Age	1.79	0.95	2.63	<0.001
Sex (male)	4.77	0.67	8.88	0.02
Athlete	21.72	17.62	25.81	<0.001

E-cigarette: electronic cigarette; IQOS: I Quit Ordinary Smoking.

**Table 8:** Multivariable linear regression models with adjustment for FEV_1_/FVC (% of predicted) R^2^ = 0.365.

Independent Variable	Beta	95% CI Low	95% CI High	*p*
Smoker/Non-smoker	−1.96	−6.03	2.11	0.34
Pack years	−0.56	−1.18	0.05	0.07
Traditional cigarette	−1.98	−5.60	1.63	0.28
E-cigarette/IQOS	2.69	0.05	5.34	0.04
Age	−0.03	−0.38	0.31	0.84
Sex (male)	−3.42	−5.15	−1.69	<0.001
Athlete	6.08	4.35	7.81	<0.001

E-cigarette: electronic cigarette; IQOS: I Quit Ordinary Smoking.

**Table 9:** Multivariable linear regression models with adjustment for FEV_1_ (% of predicted), R^2^ = 0.459.

Independent Variable	Beta	95% CI	*p*
Smokers/Non-smokers	−3.26	−10.00 to 3.47	0.34
Pack-years (/1)	−1.02	−2.04 to 0.00	0.05
Traditional cigarette (yes or no)	−3.14	−9.13 to 2.84	0.30
E-cigarette/IQOS (yes)	5.05	0.67 to 9.42	0.02
Age (/1 year)	0.49	−0.09 to 1.07	0.10
Sex (male vs. female)	−0.87	−3.74 to 1.99	0.54
Athlete (yes vs. no)	15.14	12.28 to 18.00	<0.001

E-cigarette: electronic cigarette; IQOS: I Quit Ordinary Smoking.

**Table 10:** Multivariable linear regression models with adjustment for MEF25 (%) R^2^ = 0.483.

Independent Variable	Beta	95% CI Low	95% CI High	*p*
Smoker/Non-smoker	−7.13	−16.42	2.16	0.13
Pack years	−0.95	−2.37	0.45	0.18
Traditional cigarette	−5.48	−13.74	2.77	0.19
E-cigarette/IQOS	7.32	1.28	13.35	0.01
Years	1.12	0.31	1.93	0.00
Sex (male)	2.04	−1.90	6.00	0.30
Athlete	20.76	16.81	24.71	<0.001

E-cigarette: electronic cigarette; IQOS: I Quit Ordinary Smoking.

**Table 11:** Multivariable linear regression models with adjustment for MEF50 (%) R^2^ = 0.454.

Independent Variable	Beta	95% CI Low	95% CI High	*p*
Smoker/Non-smoker	−15.94	−27.07	−4.81	0.00
Pack years	0.33	−1.35	2.03	0.69
Traditional cigarette	−7.63	−17.52	2.25	0.13
E-cigarette/IQOS	7.56	0.33	14.78	0.04
Age	0.75	−0.20	1.72	0.12
Sex (male)	4.62	−0.10	9.36	0.05
Athlete	21.56	16.84	26.29	<0.001

E-cigarette: electronic cigarette; IQOS: I Quit Ordinary Smoking.

**Table 12:** Multivariable linear regression models with adjustment for MEF75 (%) R^2^ = 0.382.

Independent Variable	Beta	95% CI Low	95% CI High	*p*
Smoker/Non-smoker	−13.81	−28.71	1.08	0.06
Pack years	−0.74	−3.01	1.52	0.51
Traditional cigarette	−11.92	−25.17	1.31	0.07
E-cigarette/IQOS	7.61	−2.06	17.29	0.12
Years	−0.52	−1.81	0.77	0.42
Sex (male)	−2.70	−9.04	3.63	0.40
Athlete	21.81	15.48	28.14	<0.001

E-cigarette: electronic cigarette; IQOS: I Quit Ordinary Smoking.

**Table 13:** Multivariate logistic regression—distal obstruction (distal DVO ≥ 1) R^2^ = 0.519.

Independent Variable	OR	95% CI	*p*
Smoker (yes vs. no)	4.86	0.87–27.12	0.07
Pack years (per 1)	1.31	1.00–1.71	0.04
Traditional cigarette (yes vs. no)	2.83	0.64–12.52	0.16
E-cigarette/IQOS (yes vs. no)	0.54	0.18–1.58	0.26
Years (/1 year)	1.01	0.83–1.22	0.91
Sex (male vs. female)	0.96	0.38–2.38	0.93
Athlete (yes vs. no)	0.03	0.01–0.10	<0.001

E-cigarette: electronic cigarette; IQOS: I Quit Ordinary Smoking.

## Data Availability

The original contributions presented in the study are included in the article, further inquiries can be directed to the corresponding author.

## Abbreviations

FVC-	Forced vital capacity.
FEV1-Forced	expiratory volume in 1 s.
FEV_1_/FVC	ratio- (Tiffeneau Index).
PEF-Peak	Expiratory Flow.
OVD-	Obstructive Ventilatory Dysfunction.
MEF25-	Maximal Expiratory Flow at 25%.
MEF50-	Maximal Expiratory Flow at 50%.
MEF75-	Maximal Expiratory Flow at 75%.
IQOS-	I Quit Ordinary Smoking.
HTPs-	Heated Tobacco Products.
GOLD-Global	Obstructive Lung Disease.
